# Hypertension Medication and Medicare Beneficiaries: Prescription Drug Coverage Satisfaction and Medication Non-Adherence among Older Adults

**DOI:** 10.3390/healthcare12070722

**Published:** 2024-03-26

**Authors:** Jeong-Hui Park, Kiyoung Kim, Mar Medina, Boon Peng Ng, Matthew Lee Smith, Okeoghene Marcel Edafetanure-Ibeh, Jongwha Chang

**Affiliations:** 1 School of Public Health, Texas A&M Health Science Center, 212 Adriance Lab Rd., College Station, TX 77843, USA; jeonghuipark@tamu.edu (J.-H.P.); kkim1982@tamu.edu (K.K.); matthew.smith@tamu.edu (M.L.S.); marceloke@tamu.edu (O.M.E.-I.); 2 School of Pharmacy, University of Texas at El Paso, 1101 N campbell St., El Paso, TX 79902, USA; mimedinag17@gmail.com; 3 College of Nursing, University of Central Florida, 12201 Research Parkway, Orlando, FL 32826, USA; boonpeng.ng@ucf.edu; 4 Center for Health Equity and Evaluation Research, Texas A&M Health Science Center, 212 Adriance Lab Rd., College Station, TX 77843, USA; 5 Center for Community Health and Aging, Texas A&M Health Science Center, 212 Adriance Lab Rd., College Station, TX 77843, USA; 6 Irma Lerma Rangel School of Pharmacy, Texas A&M Health Science Center, 159 Reynolds Medical, College Station, TX 77843, USA

**Keywords:** Medicare, antihypertensives, adherence, patient factors

## Abstract

Hypertension is so prevalent and requires strict adherence to medications to prevent further disease or death, but there is no study examining factors related to prescription drug non-adherence among 65 years old and older. This study aims to assess the likelihood of medication nonadherence among patients based on factors such as age, race, and socioeconomic status, with the goal of identifying strategies to enhance medication adherence and mitigate associated health risks. Using the 2020 Medicare Current Beneficiary Survey Public Use File to represent nationwide Medicare beneficiaries (unweighted n = 3917, weighted n = 27,134,782), medication non-adherence was related to multiple independent variables (i.e., age, sex, race/ethnicity, socioeconomic status, comorbidities, insurance coverage, and satisfaction with insurance). Cross-tabulations and Wald chi-square tests were used to determine how much each variable was related to non-adherence. Multivariate logistic regression was used to examine the association between medication non-adherence and factors such as prescription drug coverage satisfaction and cost-reducing behavior. Specific trends in medication non-adherence emerged among beneficiaries. Non-adherence was higher in older adults aged 65- to 74-year-olds and those with more chronic conditions (OR = 2.24; 95% CI = 1.74–2.89). If patients were dissatisfied with the medications on the insurance formulary or struggled to find a pharmacy that accepted their medication coverage, they had worse adherence (OR = 2.63; 95% CI = 1.80–3.84). Formulary and coverage must be expanded to improve adherence to antihypertensive medications in Medicare beneficiaries. Older adults aged 65 to 74 years may be less adherent to their medications because they do not see the seriousness of the disease and could benefit from further counseling. Patients with limited activities of daily living and more comorbidities may struggle with complex treatment regimens and should use adherence assistance tools.

## 1. Introduction

Controlling hypertension is a vital health concern because uncontrolled hypertension is a risk factor for premature death [1]. In the United States, less than one-quarter of those diagnosed with hypertension have the disease under control [2]. Hypertension costs the nation USD 131 billion a year and can lead to stroke and heart disease, which are the leading causes of death in the US [2]. In 2021, it was estimated that 64% of Medicare-fee-for-service claims were for hypertension [3]. The prevalence of hypertension among Medicare beneficiaries increases with age, with the highest prevalence occurring among those over 85 years old [3]. Further, Black, Native Alaskan, and American Indian individuals have a higher prevalence than White patients [3,4]. Similarly, preventable hospitalizations for hypertension and hospitalizations for a hypertensive emergency are higher in Black patients with Medicare than in White patients [5,6]. Looking at geographic trends, the Southern US and rural areas have some of the highest prevalence for hypertension compared to the rest of the US and urban areas, respectively [7].

Consistent adherence to antihypertensive medications is crucial because lower adherence increases the risk of stroke, heart attack, heart failure, and death [8]. Adherence can lead to better blood pressure control and improved mortality rates, but adherence to prescribed medications is low [8]. About 31% of US adults were non-adherent to their antihypertensive medications in 2015 [9]. A previous study that sampled the number of patients with Medicare who discontinued or had low adherence to their antihypertensive medications found that discontinuation rates were about 21% [10]. Patients with Medicare tend to be more adherent to their antihypertensive medications than patients with Medicaid or private insurance [9]. However, about a quarter of patients with Medicare Part D are non-adherent [9].

Adherence is related to various patient and insurance factors. In a small survey of Medicare beneficiaries with chronic diseases, the most common reason for non-adherence was forgetfulness, followed by side effects, then feeling the medication was unnecessary [11]. Low adherence rates among patients with Medicare are especially concerning in racial and ethnic minorities compared to White patients, with studies showing that Black and Hispanic patients are significantly less likely to be adherent than White patients [10,12,13,14,15,16]. During the COVID-19 pandemic, patients with Medicare were allowed to fill their medications early and for larger amounts, which helped maintain adherence levels but did not improve the disparity between White and minority patients [17]. Men tend to be more non-adherent than women [16]. Restrictive formularies that reduce drug coverage and increase out-of-pocket costs can also lead to non-adherence among older adults [18]. Greater coverage promotes medication adherence among patients with Medicare which can stave off increased costs from further complications and acute cardiovascular diseases [14,18,19,20,21].

Since hypertension is so prevalent and requires strict adherence to medications to prevent further disease or death, it is highly relevant to study factors related to prescription drug non-adherence. With over 65 million Americans using Medicare, this population requires dedicated efforts to improve adherence rates and prevent further comorbidity. This study aims to investigate factors associated with increased medication non-adherence among Medicare beneficiaries with hypertension. Relevant factors include age, sex, race/ethnicity, socioeconomic status, comorbidities, insurance coverage, and satisfaction with insurance.

## 2. Methods

### 2.1. Study Design

A retrospective study using secondary data for a cross-sectional analysis was adopted in this study. This study utilized the 2020 Medicare Current Beneficiary Survey Public Use File (MCBS PUF). The MCBS PUF is a publicly accessible version of the MCBS that includes only Medicare beneficiaries who reside in the community [2]. By using the sampling methodology described in another study [22], the MCBS becomes representative of the Medicare population. The data set includes beneficiary sociodemographic characteristics, health conditions, utilization, satisfaction with health care services, and medication-related information.

It is notable that the Centers for Medicare & Medicaid Services (CMS) and the National Opinion Research Center (NORC) implemented significant modifications to the Medicare Current Beneficiary Survey (MCBS) data collection process to ensure the safety of both respondents and field interviewers in response to the COVID-19 pandemic and its unprecedented challenges. Traditionally conducted through in-person interviews, the data collection method for both the Community and Facility portions of the MCBS was transitioned to telephone interviews starting in March 2020 [23].

### 2.2. Study Participants

Our study sample was Medicare beneficiaries aged 65 years and older with hypertension (n = 3917). Medicare recipients were asked whether a physician informed them that they had hypertension. Those who reported having hypertension were then questioned about their specific type of hypertension. This study used de-identified and publicly available data and did not require direct informed consent from participants, as it was considered non-human subjects’ research. Therefore, it was approved for a waiver by the Institutional Review Board of Texas A&M University (IRB2023-0268).

In this study, the initial inclusion criterion for participants was being aged 65 years and older, reflecting our focus on the Medicare-eligible population, which traditionally begins at 65. To further refine our analysis within this broad age group, we categorized participants into more specific age groups (65–74 and ≥75 years old). This categorization was driven by the recognition that medication adherence and the factors influencing it could vary significantly across different stages of older adulthood.

### 2.3. Measures

#### 2.3.1. Dependent Variables

In order to measure medication non-adherence during the last year, the dependent variable comprised two inquiries pertaining to the conduct of the beneficiaries. (1) “Have you missed doses regularly, occasionally, or never in an attempt to extend the duration of the medication?” (2) “Have you ever attempted to extend the duration of a drug by taking smaller-than-recommended amounts, either regularly, sometimes, or never?”

Previous studies employed similar measures and methods to ascertain instances of drug non-adherence [24]. A binary indicator of medication non-adherence was constructed, wherein those responding “sometimes” or “often” to both inquiries were assigned a value of 1, and those answering “never” to both inquiries were assigned a value of 0. To enhance the reliability of our analysis, we constructed and analyzed distinct binary dependent variables for medication non-adherence and taking smaller doses. Specifically, we accounted for the frequency or occasionality of dose skipping (1 = frequently or occasionally skipped doses; 0 = never skipped doses) and taking smaller doses (1 = frequently or occasionally taken smaller doses; 0 = never taken smaller doses).

#### 2.3.2. Independent Variables

As independent variables, beneficiaries’ satisfaction with prescription drug coverage, cost-cutting behavior, sociodemographic and health-related factors, and prescription drug coverage satisfaction were all included.

#### 2.3.3. Prescription Drug Insurance Coverage

A binary variable was generated for each of the three prescription insurance plans (private, Medicare Part D, and Medicare Advantage (Part C)) to denote whether beneficiaries were covered by that plan for prescription drugs. Briefly, private plans refer to non-governmental prescription drug coverage through employer-sponsored or self-purchased insurance. Medicare Part D is a government program offering outpatient prescription drug coverage to Medicare beneficiaries, with plans varying in coverage and costs. Medicare Advantage (Part C) plans, provided by private companies approved by Medicare, offer combined health and prescription drug coverage, often with additional benefits.

#### 2.3.4. Prescription Drug Coverage Satisfaction

A satisfaction rating with the amount paid for drugs, the quality of pharmaceuticals on the formulary, and the ease of locating a pharmacy that accepted medication coverage were the three aspects of drug insurance coverage that were assessed using three distinct criteria. The first scoring system utilized for all three survey inquiries was a four-point Likert scale. To guarantee an adequate sample size, the responses were dichotomized into two distinct groups. For the amount paid for prescription drugs, responses indicating ‘Satisfied’ or ‘Extremely satisfied’ (coded as 1 or 2 in the survey) were categorized as 0 to denote satisfaction, while responses indicating ‘Unsatisfied’ or ‘Extremely unsatisfied’ (coded as 3 or 4) were categorized as 1 to denote unsatisfaction.

#### 2.3.5. Sociodemographic and Health-Related Covariates

Inclusion criteria comprised the subsequent sociodemographic variables as follows: age range of 65 to 74 years and 75 years old and older; gender (male and female); racial composition including White, Black, Hispanic, and other; marital status including married, widowed, divorced or separated, and never married; educational attainment including high school or vocational, high school, and beyond high school; income levels including USD 10,000 and more than USD 25,000; and dwelling areas (metro, non-metro). Physical and functional limitations, the number of self-reported chronic conditions (one, two to three, and four or more), and general health status (excellent/very good, good, and fair/poor) were also incorporated as health-related covariates (no functional limitations, instrumental activities of daily living [IADLs] only, 1 to 2 activities of daily living [ADLs], and 3 or more ADLs).

### 2.4. Statistical Analysis

We ascertained the degree of variation across prescription drug coverage plans, sociodemographic and health-related factors, and medication non-adherence by employing cross-tabulations and Wald Chi-Square testing. Utilizing a multivariate logistic regression model and adjusting for sociodemographic and health-related variables, we examined the relationship between medication non-adherence and variables including satisfaction with prescription drug coverage and cost-reducing behavior. The findings of descriptive and logistic regression were weighted to reflect the Medicare population using survey weights extracted from the MCBS PUF data. Every analysis was performed utilizing version 9.4 of SAS (SAS Institute) with a *p*-value of less than 0.05.

## 3. Results

Table 1 outlines the study sample’s sociodemographic characteristics and prescription drug coverage plans according to medication non-adherence. In our study population of Medicare beneficiaries with hypertension, 11.8% (representing an estimated 3,193,687 Medicare beneficiaries) reported non-adherence to prescribed medications. Medication non-adherence showed a higher percentage among the participants aged 65–74 years old than those who adhered to their prescriptions (67.4% vs. 58.2%; *p* < 0.001). A considerable number of individuals were in inferior health compared to those who followed prescribed treatment regimens. For example, greater percentages of beneficiaries who failed to comply with their prescription regimen showed several restrictions in activities of daily living (three ADLs; 10.8 percent vs. 5.7 percent; *p* < 0.001) and a fair to poor overall health condition (28.4 percent vs. 16.4 percent; *p* < 0.01).

Compared to 55.8%, 74.2%, and 18.6% of those who adhered to their medications, 52.8%, 82.6%, and 10.8% of Medicare beneficiaries with medication non-adherence reported having no Medicare Advantage plans, no private plans, and no Part D, respectively (Table 1). In Medicare Advantage and private plans, the proportions of beneficiaries with and without medication non-adherence were not significantly different. Among those with Part D coverage, the difference between the proportions of beneficiaries with and without medication non-adherence was significant (*p* < 0.001). 

Table 2 summarizes the outcomes of the multivariate logistic regression analysis. Medicare beneficiaries under the ages 65–74 were more likely to report medication non-adherence than beneficiaries over the age of 75 (odds ratio [OR] = 2.24; 95% confidence interval [CI] = 1.74–2.89). In comparison to those who had fewer chronic conditions, beneficiaries with more chronic conditions (>4) were more likely to report medication non-adherence (OR= 2.81; 95% CI = 1.82–4.35). Medication non-adherence was linked to a higher likelihood of dissatisfaction with the cost of the medication (OR = 3.39; 95% CI = 2.43–4.72). Additionally, a higher likelihood of medication non-adherence was linked to dissatisfaction with the medications on the formulary (OR = 2.63; 95% CI = 1.80–3.84). Finally, there was a stronger correlation between ADL limitation (only 1 ADL limited) and medication non-adherence (OR = 1.85; 95% CI = 1.34–2.57).

## 4. Discussion

This study examined hypertension medication non-adherence among Medicare beneficiaries in an attempt to identify opportunities to promote adherence. This study found that most non-adherent patients were aged 65–74 years. Income, comorbidities, health status, drug coverage, and satisfaction were also significantly associated with adherence, but race, marital status, and education were not. These findings were repeated in the survey-weighted model, except income and drug coverage were no longer statistically significant. 

In the current study, patients 65–74 years old are more likely to be non-adherent to their antihypertensive medications compared to patients over 75 years of age. These results mirror previous research reporting that older patients aged 65 to 74 years were more non-adherent than older patients aged 75 and older [9,25,26]. Some have postulated that older patients (75+ year-olds) are more adherent than patients aged 65 to 74 years old because they are more aware of their disease severity and health, motivating them to be more adherent [26]. However, since lower blood pressure decreases the risk of stroke, coronary heart disease, and heart failure [27], it is still essential all Medicare beneficiaries understand the importance of using their antihypertensive medications. This could be a potential area for greater patient education and counseling to ensure adherence and understanding of their condition.

Comorbidities and health status were also statistically significant, with the risk of non-adherence increasing as the number of chronic conditions increased. Having more than four chronic conditions was associated with the greatest risk of non-adherence. This finding is similar to past research that found that increased comorbidities and polypharmacy are negatively associated with adherence [12,26]. Similarly, limitations to activities of daily living increase the risk of non-adherence. These two findings coincide in that an increased pill burden or treatment complexity and limitations to daily living are obvious factors that can impair adherence. However, they are both factors that medication assistance tools can significantly impact to make taking and remembering medications easier [28].

Greater dissatisfaction with the amount paid for and the medications on the formulary significantly increased the risk of non-adherence in the present study. Cost and formulary restrictions have previously been found to impact adherence negatively [18]. Overcoming either issue is more complicated as it involves improved drug coverage, expanded formularies, and decreased out-of-pocket costs. Advancements to improve access are still preferable to the increased costs stemming from later hospitalizations and worsening comorbidities [14,18,19,20,21].

This study is subject to several potential limitations. Initially, it is essential to note that medication non-adherence levels were self-reported due to the lack of access to claims data within the MCBS PUF. This approach may have introduced recall and social desirability biases, as suggested by most scholarly articles, which could have resulted in overestimating adherence [29,30]. Although medication possession rate, the proportion of days covered, and claims data can be utilized in a non-adherence analysis to measure refill behavior accurately, this does not guarantee an accurate representation of actual medication intake or adherence to prescribed regimens. Non-adherence behavior, such as forgoing the prescribed medication or failing to take it as directed, could be more accurately captured through self-reported non-adherence. Secondly, the current study’s adherence rate may be overestimated due to the possibility that beneficiaries classified as adherents did not require any medication. However, this is improbable given that the study population consisted of individuals with hypertension. Finally, due to the MCBS PUF’s cross-sectional nature, it is impossible to establish a causal relationship between the associated factors.

## 5. Conclusions

This study underscores the critical importance of addressing non-adherence among Medicare beneficiaries with hypertension. The findings suggest that enhancing patient adherence to prescribed medications and treatment regimens can significantly improve hypertension control, ultimately reducing the risk of further illness, hospitalizations, and mortality. To achieve this goal, healthcare providers and policymakers must collaborate to implement multifaceted solutions such as enhanced counseling, expanded formulary and coverage options, and adherence assistance tools. Also, Medicare beneficiaries may benefit from enhanced counseling, expanded formulary and coverage, and adherence assistance tools to improve adherence and prevent further illness. These solutions may seem simple but require interprofessional collaboration to overcome barriers to access and adherence. 

## Figures and Tables

**Table 1:** Characteristics of Medicare beneficiaries aged ≥65 years with hypertension by medication non-adherence (total).

Variable	Total	Medication Adherence	Medication Non-Adherence	*p*-Value
Unweighted n	3917	3497	420	
Weighted n	27,134,782	23,941,094	3,193,687	
Overall (weighted %)	100	88.2	11.8	
**Sociodemographic Characteristics**				
Age group				<0.001
65–74 years	55.8	54.2	67.4	
≥75 years	44.2	45.8	32.6	
**Sex**				0.058
Female	57.2	56.6	62.0	
Male	42.8	43.4	38.0	
**Race/ethnicity**				0.153
White	75.4	76.0	70.7	
Black	10.6	10.3	12.8	
Hispanic	7.6	7.7	6.8	
Other	6.5	6.0	9.6	
**Marital status**				0.731
Married	54.6	54.9	52.6	
Widowed	23.5	23.5	23.8	
Divorced/separated	15.9	15.8	16.3	
Never married	6.0	5.8	7.3	
**Education**				0.267
Less than HS	11.9	11.7	13.1	
HS or vocational	30.0	29.6	32.7	
More than HS	58.1	58.6	54.1	
**Income**				<0.001
<USD 25,000	26.8	25.2	34.4	
≥USD 25,000	73.2	74.8	65.6	
**Residing area**				0.181
Non-metro area	16.6	16.3	19.1	
Metro	83.4	83.7	80.9	
**Comorbidities and Health Status**				
**Number of chronic conditions**				<0.001
0–1	30.5	32.5	15.3	
2–3	50.1	50.2	50.0	
≥4	19.4	17.4	34.7	
**ADL limitations**				<0.001
No limitations	63.7	66.3	43.9	
Only IADLs limited	12.6	11.6	19.9	
1–2 ADLs limited	17.4	16.3	25.4	
≥3 ADLs limited	6.3	5.7	10.8	
**General health status**				<0.001
Excellent/very good	50.5	52.8	33.9	
Good	31.7	30.9	37.8	
Fair/poor	17.8	16.4	28.4	
**Drug Coverage Plan**				
**Medicare Advantage plan coverage**				0.443
No	55.5	55.8	52.8	
Yes	44.5	44.2	47.2	
**Private plan coverage**				0.003
No	75.2	74.2	82.6	
Yes	24.8	25.8	17.4	
**Part D plan coverage**				<0.001
No	17.6	18.6	10.8	
Yes	82.4	81.4	89.2	
**Prescription Drug Insurance Satisfaction**				
**Dissatisfaction: the amount paid** **for medications**				<0.001
No	90.0	92.6	70.9	
Yes	10.0	7.4	29.1	
**Dissatisfaction: the medications on the formulary**				<0.001
No	95.0	96.5	83.0	
Yes	5.1	3.5	17.0	
**Dissatisfaction: find a pharmacy that accepted** **medication coverage**				0.039
No	98.9	99.1	97.0	
Yes	1.1	0.9	3.0	

Weighted n represents the estimated number of Medicare beneficiaries based on the MCBS recommended analytical method and weight variable used.

**Table 2:** Survey-weighted logistic model to predict factors associated with medication non-adherence among Medicare beneficiaries aged ≥65 years with hypertension.

Variable	OR *	95% CI ^†^	*p*-Value
**Sociodemographic Characteristics**			
**Age group**			
65–74 years	2.24	1.74–2.89	<0.001
≥75 years	Ref ^‡^		
**Sex**			
Female	Ref		
Male	0.94	0.72–1.24	0.663
**Race/ethnicity**			
White	Ref		
Black	1.04	0.65–1.67	0.869
Hispanic	0.74	0.42–1.33	0.310
Other	1.81	1.08–3.02	0.025
**Marital status**			
Married	Ref		
Widowed	1.02	0.73–1.41	0.926
Divorced/separated	0.86	0.59–1.25	0.411
Never married	1.18	0.72–1.92	0.514
**Education**			
Less than HS	0.75	0.47–1.20	0.233
HS or vocational	Ref		
More than HS	0.92	0.72–1.19	0.537
**Income**			
<USD 25,000	1.38	0.97–1.98	0.077
≥USD 25,000	Ref		
**Residing area**			
Non-metro area	Ref		
Metro	1.08	0.83–1.40	0.578
**Comorbidities and Health Status**			
**Number of chronic conditions**			
0–1	Ref		
2–3	1.81	1.37–2.39	<0.001
≥4	2.81	1.82–4.35	<0.001
**ADL limitations**			
No limitations	Ref		
Only IADLs limited	1.85	1.34–2.57	<0.001
1–2 ADLs limited	1.62	1.13–2.31	0.010
≥3 ADLs limited	1.84	1.16–2.92	0.010
**General health status**			
Excellent/very good	0.69	0.51–0.95	0.023
Good	Ref		
Fair/poor	0.81	0.54–1.21	0.294
**Drug Coverage**			
**Medicare advantage plan coverage**			
No	Ref		
Yes	1.06	0.78–1.44	0.716
**Private plan coverage**			
No	Ref		
Yes	0.75	0.53–1.08	0.126
**Part D plan coverage**			
No	Ref		
Yes	1.32	0.81–2.15	0.255
**Prescription Drug Insurance Satisfaction**			
**Dissatisfaction: the amount paid for medications**			
No	Ref		
Yes	3.39	2.43–4.72	<0.001
**Dissatisfaction: the medications on the formulary**			
No	Ref		
Yes	2.63	1.80–3.84	<0.001
**Dissatisfaction: find a pharmacy that accepted** **medication coverage**			
No	Ref		
Yes	2.01	0.84–4.77	0.114

* OR: Odds ratio; ^†^ CI: Confidence interval; ^‡^ Ref: Reference group.

## Data Availability

The datasets used and/or analyzed during the current study are available from the corresponding author upon reasonable request.
